# Visualising and Thinking and Interpreting. Response to the Burstyn and De Roos Comments on Sorahan, T. Multiple Myeloma and Glyphosate Use: A Re-Analysis of US Agricultural Health Study (AHS) Data. *Int. J. Environ. Res. Public Health* 2015, *12*, 1548–1559

**DOI:** 10.3390/ijerph14010006

**Published:** 2016-12-22

**Authors:** Tom Sorahan

**Affiliations:** Institute of Applied Health Research, University of Birmingham, Edgbaston, Birmingham B15 2TT, UK; t.m.sorahan@bham.ac.uk; Tel.: +44-121-414-3644

I am grateful to the Editor for the opportunity of responding to the recent paper of Burstyn and De Roos [1], that is in part, a critical commentary of my own analysis of data from the US Agricultural Health Study (AHS) concerning the findings for multiple myeloma and glyphosate use [2].

Firstly, I agree with Burstyn and De Roos that the more important findings from any epidemiological study are those relating to levels of estimated or measured exposure (so-called dose-response analyses) rather than simple ever/never exposed comparisons. That is why, in my own analyses, I showed the results of eight dose-response analyses; none was statistically significant and most were a long way from being statistically significant. So why did I also put some considerable effort into working out whether there was any basis for preferring one of the two findings supplied by De Roos et al. [3] for ever-use of glyphosate? [3]: Rate ratio (RR) of 1.1, 95% Confidence Interval (CI) 0.5 to 2.4, in full dataset adjusted for age only; RR of 2.6, 95% CI 0.7 to 9.4, in restricted dataset with adjustment for many variables. It is because one could be reasonably confident that one or other of these values would be used in future meta-analyses that would, by necessity, be limited to making use of these simplistic overall findings. It would, therefore, be important to know which estimate could be relied upon. I have checked the logic and language in my original paper and my conclusion that the risk estimate of 2.6 arose from the use of a restricted dataset that, probably by chance, turned out to be unrepresentative, is correct, polite and fair. Burstyn and De Roos [1] would appear to agree with this when they state ‘there is likely selection bias adversely affecting the analysis with ever- vs. never-exposed.’

Burstyn and De Roos [1] are concerned about relying on ‘intuition’, and sole reliance on any single skill or aptitude is probably dangerous. But on first reading of the De Roos et al. paper [3] more than ten years ago, I strongly suspected that something very odd must be going on to produce such disparate findings for ever-use of glyphosate. Whether that is intuition or experience is a moot point.

Finally, I fully agree with Burstyn and De Roos [1] that an updated AHS needs to be analysed. The pesticide applicators that are participating in this key survey are stakeholders. Can’t they make representations to bring such an analysis about?
